# Autoimmune gastritis as an unexpected cause of diarrhea in a young adult with type I diabetes: a case report

**DOI:** 10.1186/s13256-023-04039-0

**Published:** 2023-07-29

**Authors:** Taylor M. Meyers, Patrick T. Reeves, Jamie L. Lombardo, Sarah K. Anisowicz, Noelle S. Larson, Philip L. Rogers

**Affiliations:** 1 Department of Pediatrics, Walter Reed National Military Medical Center, 4494 Palmer Road North, Bethesda, MD 20814 USA; 2 Department of Pediatrics, Uniformed Services University of the Health Sciences, Bethesda, MD USA

**Keywords:** Autoimmune gastritis, Autoimmunity, Diabetes mellitus type 1, Vitamin B12 deficiency, Case report

## Abstract

**Background:**

Type 1 diabetes mellitus (T1DM) is a lifelong diagnosis that involves immune-mediated damage of pancreatic beta cells and subsequent hyperglycemia, manifesting as: polyuria, polydipsia, polyphagia, and weight loss. Treatment of type 1 diabetes centers on insulin administration to replace or supplement the body's own insulin with the goal of achieving euglycemia and preventing or minimizing complications. Patients with T1DM are at risk for developing other autoimmune conditions, most commonly thyroid or celiac disease.

**Case presentation:**

A 20-year-old African American female with T1DM was referred by her endocrinologist to pediatric gastroenterology for 2 months of nocturnal, non-bloody diarrhea, left lower quadrant pain, and nausea; she was also being followed by neurology for complaints of lower extremity paresthesias and pain. The patient’s initial lab-workup was remarkable for a low total Immunoglobulin A (IgA) level of < 6.7 mg/dL. As IgA deficiency is associated with an increased risk of celiac disease, the patient underwent upper and lower endoscopy, which was grossly unremarkable; however, histology revealed a pattern consistent with autoimmune gastritis. Subsequent serum evaluation was remarkable for an elevated fasting gastrin level and an elevated parietal cell antibody level without macrocytic anemia, iron deficiency, or vitamin B12 depletion. The patient was diagnosed with autoimmune gastritis (AIG) and subsequently initiated on parenteral B12 supplementation therapy with improvement in her neurologic and gastrointestinal symptoms.

**Conclusion:**

This case illustrates the importance of recognition of red flag findings in a patient with known autoimmune disease. Following well-established health maintenance recommendations for individuals with T1DM ensures that common comorbidities will be detected. Autoimmune gastritis, while a rarer pathology in the pediatric population, deserves consideration in patients with pre-existing autoimmune conditions and new gastrointestinal or neurologic symptoms, as AIG can be associated with poor outcomes and risk of malignancy. Initial lab findings associated with an eventual diagnosis of AIG typically include anemia, iron deficiency, or Vitamin B12 deficiency. However, as demonstrated in this case, symptoms of AIG can rarely present before anemia or Vitamin B12 deficiency develops. To prevent permanent neurological damage, parenteral Vitamin B12 therapy must be considered even in the absence of Vitamin B12 deficiency, especially in those patients already experiencing neurological symptoms.

## Background

Type 1 diabetes mellitus (T1DM) involves immune-mediated damage of pancreatic beta cells and subsequent hyperglycemia, manifesting as: polyuria, polydipsia, polyphagia, and weight loss [1].

Lifelong management of T1DM includes lifestyle changes (such as initiation of regular exercise and adjustment of diet, if needed) and education on diabetes self-management; the most important component of self-management includes close serum glucose monitoring and aggressive insulin therapy with a combination of either bolus and basal insulin injections or continuous insulin infusion through an insulin pump. Maintaining tight glycemic control can help prevent complications of chronic hyperglycemia (such as those related to macrovascular and microvascular damage: heart disease, stroke, diabetic retinopathy, nephropathy, etc.) [2]. In caring for a patient with T1DM, the physician must adhere to chronic health maintenance guidelines, with specific focus on history and physical exam, immunizations, laboratory studies, and promotion of diabetes self-management and education. For example, unique health maintenance recommendations for children with longstanding T1DM include: annual foot and dilated ocular exams. Laboratory testing includes: quarterly Hemoglobin A1c (HgbA1c), annual creatinine and urine albumin-to-creatinine ratio [2].

In addition to the aforementioned complications from chronic hyperglycemia, patients with T1DM are also at risk for developing other autoimmune conditions, most commonly thyroid or celiac disease, as there is an established association between T1DM and other autoimmune diseases, likely related to shared genetic susceptibility loci and environmental exposure [3]. Thyroid antibody testing should be performed at diagnosis followed by annual thyroid stimulation hormone testing [3]. Celiac disease screening at diagnosis must include a tissue transglutaminase Immunoglobulin (IgA) level and a total IgA level, because selective IgA deficiency increases the risk for celiac disease [4].

## Case presentation

A previously healthy 20 year old African American female with T1DM (diagnosed at age 10, last HgbA1c = 7.8%) is referred to Pediatric gastroenterology for 2 months of nocturnal, non-bloody diarrhea, left lower quadrant pain, and nausea. Her review of systems was remarkable for normal daytime bowel movements. She also complained of lower extremity pains with numbness for which she had been referred to neurology for concerns of diabetic neuropathy.

Her past medical history was remarkable for T1DM without history of diabetic ketoacidosis (Anti-glutamic acid decarboxylase antibody (13.1 U/mL) and Islet Antigen-2 Autoantibody were positive at time of diagnosis), IgA deficiency, and high risk for celiac disease with HLA DQ2 heterozygosity. Family history revealed a father with hyperlipidemia and hypertension and a maternal grandfather with Type 2 Diabetes Mellitus, but otherwise no family medical history of gastrointestinal or autoimmune pathologies. Psychosocial history, including HEADSS (Home, Education, Activities, Drug/alcohol use, suicidality, and sexual activity) assessment, was reassuring. Physical exam during her pediatric gastroenterology appointment was benign, including abdominal exam. A neurological exam was performed by pediatric neurology for her symptoms of lower extremity pain and numbness and was remarkable for slightly diminished sensation to vibration and pinprick in bilateral toes, but otherwise normal motor and strength testing, reflexes, and cranial nerve function. While undergoing further work-up with pediatric gastroenterology, the patient was trialled on Bisacodyl, which did not improve her gastrointestinal symptoms. The patient’s initial lab-workup was remarkable for a low total IgA of < 6.7 mg/dL (Table 1). The selective IgA deficiency and lower tract symptoms increased the gastroenterologist’s concern for Celiac disease or ulcerative colitis. Irritable bowel syndrome was also considered as a potential diagnosis.

**Table 1:** Patient’s clinical course and lab evaluation

Date	Pertinent labs	Units	Reference range
16 February 2021 Patient sees endocrinologist, and is referred to Pediatric gastroenterology for abdominal symptoms	**HA1c 7.8**	%	4.2–6.0
	Thyroid Stimulating Hormone 2.360	mcIU/mL	0.27–4.2
	Free Thyroxine 1.20	ng/dL	0.93–1.7
	Iron panel		
	Iron 59	mcg/dL	37–145
	Total Iron Binding Capacity 368	mcg/dL	228–428
	Iron Saturation 16.1	%	15–50
	Ferritin 46	ng/mL	13–150
	Complete blood count		
	White Blood Cells 7.4	X10^3/mcL	4.2–9.4
	Red Blood Cells 4.39	X10^6/mcL	4.0–5.0
	Hemoglobin 12.3	g/dL	11.9–15.3
	**Platelets 394**	X10^3/mcL	170–388
02 March 2021 Patient sees gastroenterologist and is scheduled for upper endoscopy and colonoscopy on 09March2021	Celiac Panel		
	** Total serum IgA < 6.7**	mg/dL	44–461
30 March 2021 Patient returns to gastroenterologist, diagnosis of Autoimmune Gastritis is discussed 05 April 2021 Patient started on Vitamin B12 therapy Complimentary battlefield acupuncture is implemented to relieve remaining symptoms	Vitamin B12 (Cobalamin) 428.9	pg/mL	232–1245
	Folate 10.58	ng/mL	4.6–34.8
	Complete blood count		
	White Blood Cells 6.8	X10^3/mcL	4.2–9.4
	Red Blood Cells 4.54	X10^6/mcL	4.0–5.0
	Hemoglobin 12.7	g/dL	11.9–15.3
	**Platelets 412**	X10^3/mcL	170–388
	Intrinsic Factor Antibody 1.0	AU/mL	0.0–1.1
	**Parietal Cell Ab 104.1**	Units	0.0–20.0
	**Gastrin 1680**	pg/mL	0–115
	Fecal Calprotectin 110	mcg/g	0–120

The bolded values indicate values that are abnormal (outside of the normal reference range)

**Fig. 1:**
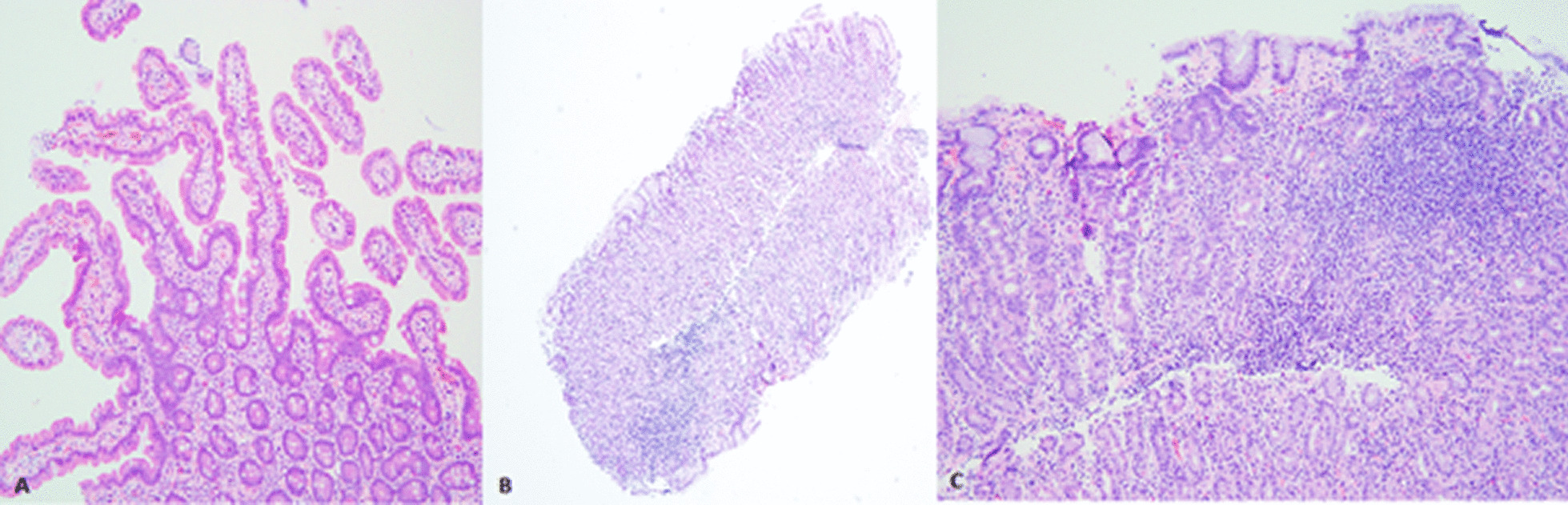
**A** Duodenal biopsy demonstrating preserved villous architecture with long villous structures. There is no increase in intraepithelial lymphocytes. **B** Gastric body biopsy with chronic lymphoplasmacytic inflammation in the lamina propria (lower left). ×50 magnification. **C** Gastric body biopsy at ×100, demonstrating increased chronic lamina propria inflammation and lack of parietal cells

The patient underwent upper and lower endoscopy which was grossly unremarkable; however histology revealed a pattern consistent with autoimmune gastritis (as demonstrated in Fig. 1). Biopsies were negative for *Heliobacter pylori*. Subsequent serum evaluation was consistent with pathologic diagnosis, demonstrating an elevated fasting gastrin level and an elevated parietal cell antibody level without macrocytic anemia or vitamin B12 depletion, further supporting a diagnosis of AIG. Celiac disease was excluded based on gold standard diagnostics, to include upper endoscopy with small bowel biopsies, which did not demonstrate the typical pathologic pattern of celiac disease (i.e. damage to or flattening of villi, increased intraepithelial lymphocytes, crypt hyperplasia).

The patient was subsequently initiated on B12 supplementation therapy, administered as a 1000 mcg injection once a week for 8 weeks, with subsequent resolution of her lower extremity symptoms, improvement in her gastrointestinal symptoms, and continued normal Vitamin B12 levels and complete blood counts. Thereafter, plans for further treatment were to be guided by the patient’s symptomatology and regular lab screening for vitamin B12 deficiency and anemia.

## Discussion and conclusions

Atrophic gastritis is a preneoplastic condition wherein normal gastric tissue is replaced by either connective tissue (nonmetaplastic atrophy) or metaplastic tissue secondary to chronic inflammation. The most common cause of atrophic gastritis is *Heliobacter pylori* infection [5]. Autoimmune gastritis is the second most common etiology of atrophic gastritis, and involves immune-mediated destruction of parietal cells in the corpus and fundus of the stomach. This reduces intrinsic factor production which can lead to vitamin B12 deficiency and pernicious anemia, and can result in neurologic symptoms: fatigue, headache, weakness; gastrointestinal symptoms: dyspepsia, epigastric pain; or other system involvement [6]. Diagnosis of AIG requires topographical biopsies of the gastric body and antrum with subsequent typical gastric histopathology findings [7]. In our patient, her gastric body biopsies were consistent with a diagnosis of AIG, as they demonstrated chronic lamina propria inflammation and lack of parietal cells. Additionally, her diagnosis was corroborated by her elevated gastrin level and presence of parietal cell antibodies.

Though our patient had elevated gastrin level and parietal cell antibodies, as well as pathologic findings consistent with AIG, she did not meet typical criteria for pernicious anemia with a normal hemoglobin, hematocrit, and mean corpuscular volume (MCV), as well as normal Vitamin B12 and iron levels. Comparatively, other case reports of pediatric patients diagnosed with AIG frequently describe laboratory findings of anemia, iron deficiency, and/or Vitamin B12 deficiency prior to AIG diagnosis [8]. With this key association in mind, a multi-disciplinary discussion between pediatric gastroenterology and neurology occurred and concluded that inflammation that can occur from autoimmune gastritis could potentially manifest as neuropathic symptoms from small nerve fiber injury prior to evidence of significant vitamin B12 depletion or anemia. Additionally, AIG impairs the patient’s ability to absorb Vitamin B12 enterally due to parietal cell loss and autoantibodies targeting Intrinsic Factor, and if untreated could result in overt Vitamin B12 deficiency and subsequent irreversible neurologic damage [7]. With the patient already demonstrating an abnormal neurological examination and experiencing neurological symptoms of numbness and pain in her lower extremities, parenteral Vitamin B12 therapy was initiated despite a normal Vitamin B12 level and absence of anemia to bypass this deficiency in enteral absorption in order to prevent development of permanent neurologic deficits. We hypothesize that this decision to supplement her Vitamin B12 levels in the absence of Vitamin B12 deficiency ultimately resulted in resolution of her lower extremity pains and numbness and improvement in her gastrointestinal symptoms. With regards to her gastrointestinal symptoms, it remains unclear if these symptoms were related to her AIG, or if the patient also has a degree of underlying irritable bowel syndrome (IBS), as nocturnal diarrhea and left lower quadrant abdominal pain are not typical symptoms of AIG, but can be associated with IBS. Additionally, constipation was also considered as a cause of left lower quadrant pain in this patient, although as previously stated her daytime bowel habits were described as normal.

Management of AIG involves treating any resultant micronutrient deficiencies from chronic gastric inflammation, monitoring for development of other autoimmune conditions, and surveilling for neoplastic complications [7]. Pediatric patients with autoimmune gastritis warrant upper endoscopy surveillance every 3 years to monitor for development of intestinal metaplasia, dysplasia, and progression to adenocarcinoma, which can occur from ongoing inflammation within the fundus. Additionally, there is also risk for transformation of enterochromaffin-like cells due to prolonged high circulating levels of gastrin, and can result in gastric neuroendocrine (carcinoid) tumors [9].

This case illustrates the importance of high suspicion for and recognition of red flag findings in a patient with known autoimmune disease. Clinicians should be aware of the lifelong risk for patients with T1DM to develop other autoimmune conditions, even pathologies as uncommon AIG (notably, the risk of AIG in patients with T1DM is elevated 3 to 5 times that of the general population [7]). Following well-established health maintenance recommendations for individuals with T1DM ensures that common comorbidities will be detected. Autoimmune gastritis must be considered in pediatric patients with pre-existing autoimmune conditions and new gastrointestinal or neurologic symptoms, as significant morbidity, and even mortality can occur if undetected, with potential for development of symptomatic macrocytic anemia from Vitamin B12 and iron depletion, as well as risk of ongoing inflammation resulting in preneoplastic and neoplastic changes within the gastrointestinal tract. It is also pertinent to remember that while uncommon, symptoms of AIG can present before typical laboratory abnormalities of anemia, iron deficiency, and Vitamin B12 deficiency develop. Due to impairment in enteral Vitamin B12 absorption in AIG and the risk for permanent neurological deficits from Vitamin B12 depletion, initiation of parenteral Vitamin B12 therapy should be considered even in the absence of Vitamin B12 deficiency. Vigilance and employing a multidisciplinary approach are essential to promptly detect rarer, but impactful associated conditions.

## Data Availability

Not applicable.

## Abbreviations

T1DM: Type 1 diabetes mellitus
AIG: Autoimmune gastritis
HgbA1c: Hemoglobin A1c
MCV: Mean corpuscular volume
IBS: Irritable bowel syndrome
